# Supplementary material for: Lesion distribution and network mapping in dyskinetic cerebral palsy
Source: Brain Commun. 2025 Jun 13;7(3):fcaf228. doi: 10.1093/braincomms/fcaf228 (PMC12199781; doi:10.1093/braincomms/fcaf228)
Supplement: fcaf228_Supplementary_Data [file fcaf228_supplementary_data.pdf]

Supplementary Material

for

Lesion distribution and network mapping in dyskinetic cerebral palsy

Ana Luísa de Almeida Marcelino†, Bassam Al-Fatly†, Mehmet Tuncer, Ingeborg Krägeloh-Mann, Anne Koy, Andrea A. Kühn

Supplementary Methods

Searching Terms for Scoping Review

a. ((cerebral palsy) OR (acquired dystonia))
b. AND (dyskinesia OR dyskinetic OR chorea OR choreiform OR dystonia OR dystonic OR athetosis OR athetotic OR athetoid OR choreoathetosis OR choreoathetoic OR choreoathetoid)
c. AND (MRI OR imaging OR neuroimaging OR lesion)
d. NOT (Review[Publication Type])

Functional parcellation of the thalamus

Since the cluster identified in lesion network mapping of dyskinetic cerebral palsy was located inside the thalamus, a post-hoc analysis to determine which functional thalamic subregion was highly connected to the lesions was necessary. To do so, we used the 100 subjects from the normative resting-state functional pediatric connectome [1] to parcellate the thalamus in the into four main functional zones, namely the motor, associated, limbic and other brain regions. First, *cortical* masks (or regions of interest) representative of the motor, associative, limbic and other brain regions were built by combining different cortical regions from the Haskins pediatric atlas [2]. No subcortical and cerebellar regions were included in the region of interest masks. In order to do so, we warped the Haskins brain template and its associated atlas to the pediatric MNI space and later selected single cortical regions to be contributing to each cortical mask. Specifically, the postcentral paracentral, and precentral gyri were merged to form the motor cortex mask [3]. The limbic cortex mask was extracted based on merging of the orbitofrontal, frontopolar cortices and the anterior cingulate gyri, while the associative cortex comprised all the remaining prefrontal cortices. All other brain regions (other parietal, temporal and occipital regions) were merged into one functional category as "others". Of note, Haskins to pediatric MNI spatial warping was carried out by linearly co-registering (using SPM[4]) and nonlinearly normalizing (using ANTs, http://stnava.github.io/ANTs/[5]) the Haskins template to the pediatric MNI template in Lead-DBS toolbox[3]. The resulting warp field was then applied to the associated Haskins annotated atlas. We then run functional connectivity from the combined cortical masks (motor, associative, limbic and others) to the rest of the brain in each of the pediatric normative connectome subjects. This yielded 100 connectivity maps from each cortical mask (in total 4 x 100 maps). The maps were then masked by a thalamic binary image extracted from the pediatric Haskins atlas. Voxels were assigned as belonging to a specific parcel using a winner takes all strategy[6]. The winner takes all method ensures that a specific thalamic voxel is highly connected to the specific cortical region across the 100 subjects of the pediatric normative connectome. The distribution of the thalamic parcels was visualized and compared to that of published adult thalamic parcellation by overlaying the final parcellation masks onto a pediatric MNI template[7] (Supplementary Figure 1). The spatial distribution of the parcels conforms to a similar spatial distribution when compared to its adult counterpart[8] (https://github.com/BrainMappingLab/Functional-territories-of-basal-ganglia). Note that the adult parcellation is based on structural imaging data.

Supplementary Figures

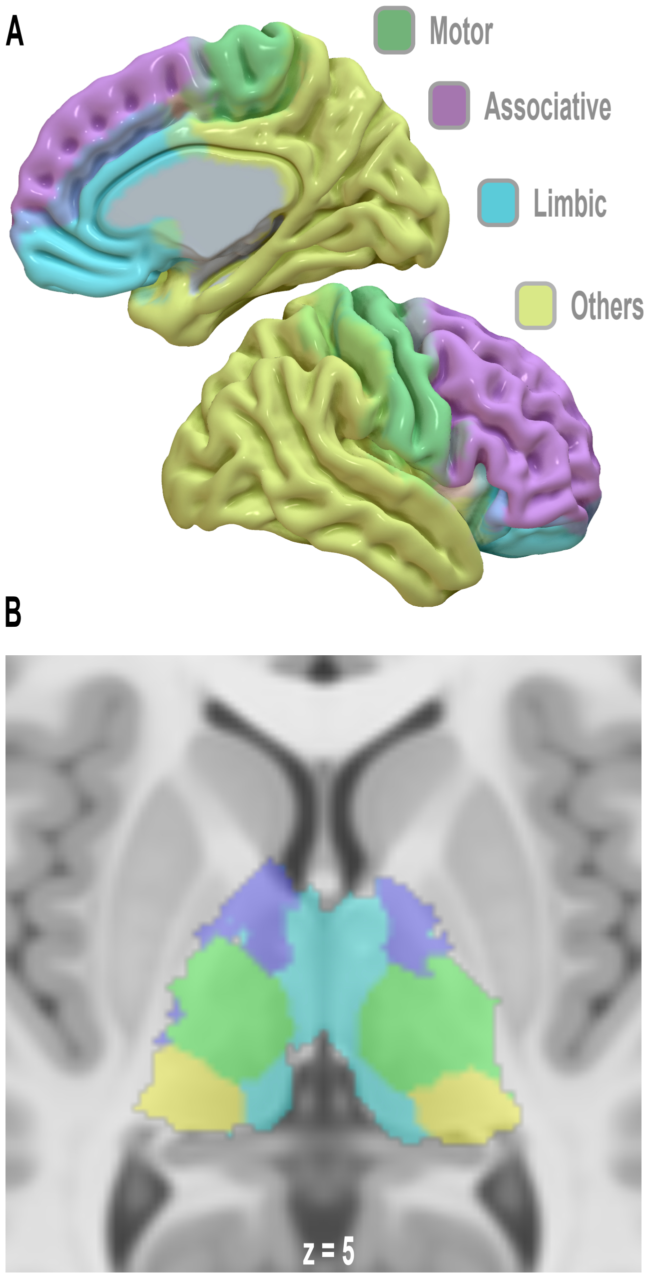

Supplementary Figure 1: Functional Parcellations of the Pediatric Thalamus. (A) Cortical functional zones were formed by merging multiple brain regions into one category. Motor (green), associative (purple), limbic (cyan) and others (yellow) masks where then used as seed regions in subject-wise connectivity mapping using the rs-fMRI acquisitions from the 100 neurotypical subjects in the pediatric connectome. The resulting maps were masked to extract only information inside thalamic voxels and later grouped into four different thalamic parcels (B) using winner takes all parcellation scheme.

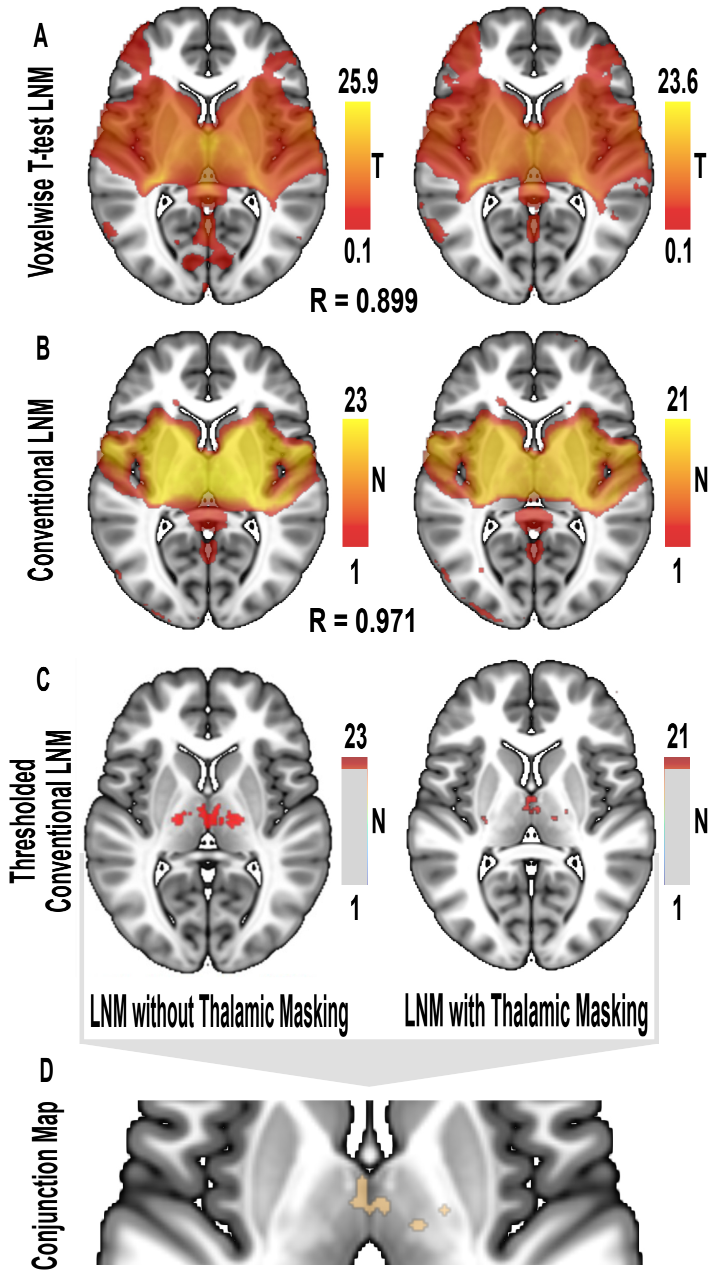

Supplementary Figure 2: LNM Control Analysis. Masking out thalamic lesions did not lead to major differences in LNM spatial distribution as demonstrated by unthresholded maps regardless of the method of lesion network mapping (voxelwise one sample t-test (A) and conventional binary overlap of T > 7 lesions associated connectivity profiles (B)). High spatial similarity was calculated between maps with and without thalamic masking using Pearson correlation in both methods and reported as R value. Panel (C) demonstrates strict thresholding of the binary overlap conventional LNM with and without thalamic masking. Conjunction of the latter maps (D) could still highlight the central thalamic cluster in the mediodorsal nucleus and the lateral cluster in the ventral intermediate nucleus.

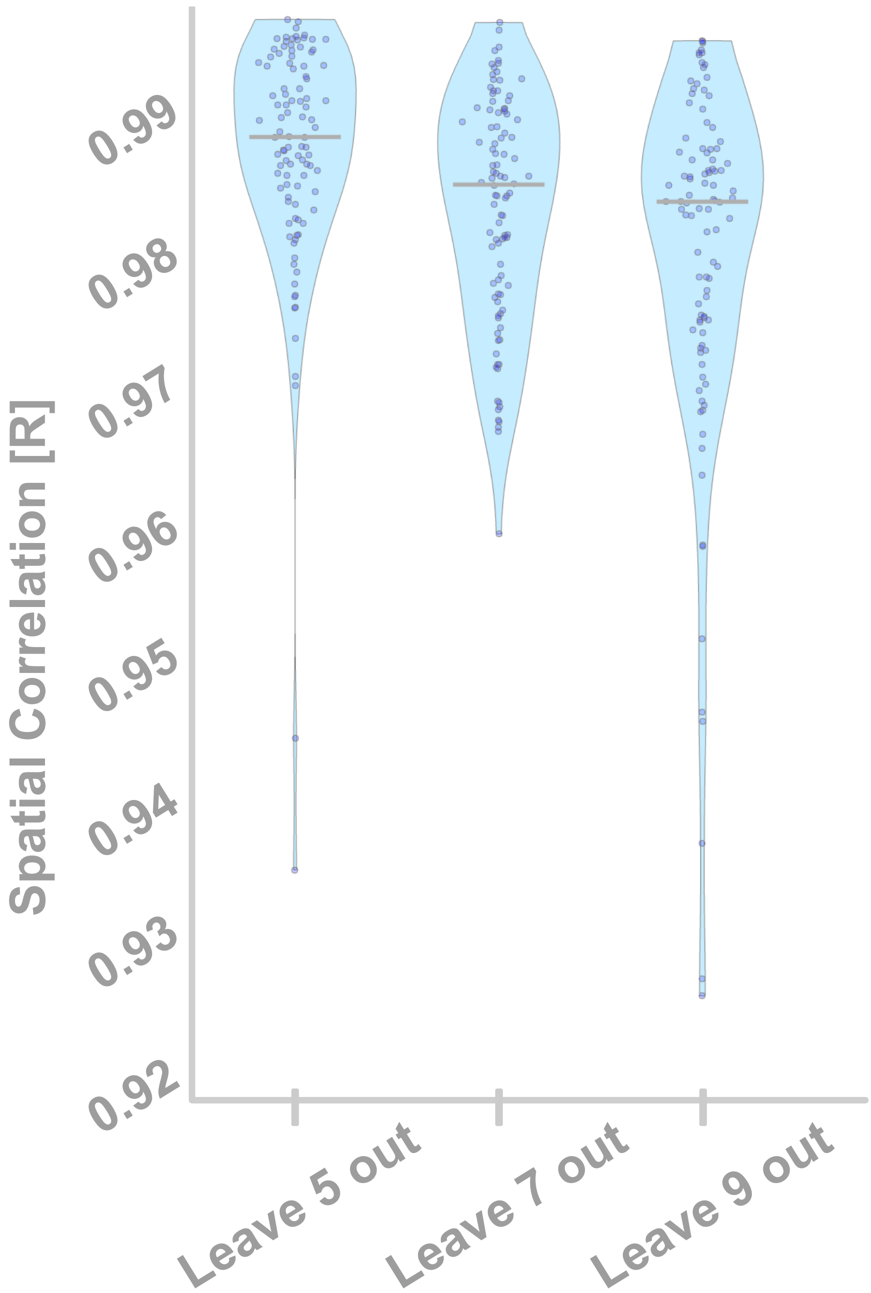

Supplementary Figure 3: Stability of the LNM. Repeating the LNM using different combination of lesion cases (n = 100 combinations) by leaving 5, 7 or 9 random cases out could still show high spatial similarity (denoted by the spatial correlation [R] on the y-axis) to the LNM including all cases. Of note, the voxelwise one-sample t-test version of the LNM has been used in this analysis as an example. Spatial similarities were also highly stable when using conventional binary overlap LNM instead (data not shown).

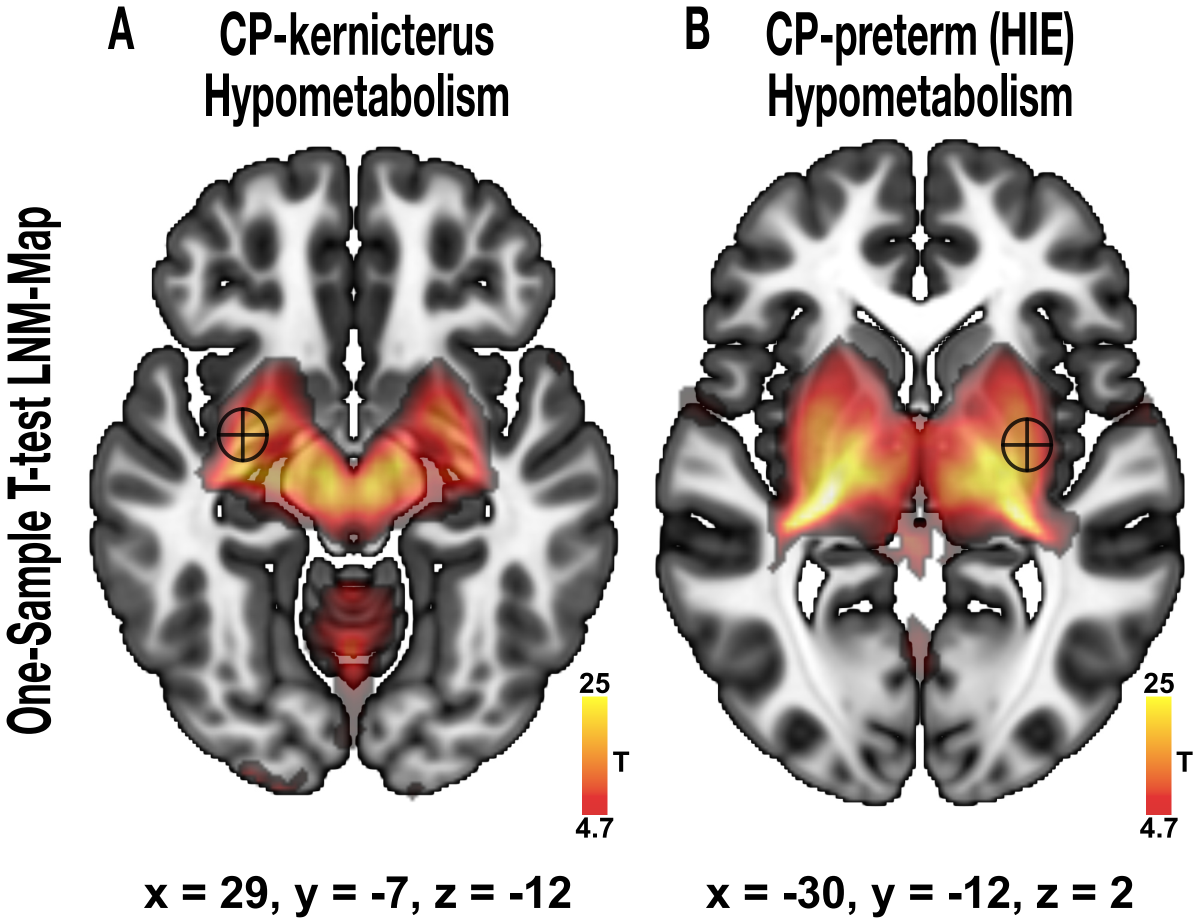

Supplementary Figure 4: Relationship of one-sample t-test LNM (from $N = 23$ cases) to hypometabolism coordinates identified by Tsagkaris and colleagues[9] using FDG-PET. Both coordinates of peak hypometabolism occurring in CP-kernicterus (A) and hypoxic ischemic encephalopathy (B) cases were depicted as a black circle in projection to the LNM results and are mentioned below each axial slice. The coordinates are provided in the supplementary material of Tsagkaris et al.[9] CP: cerebral palsy; HIE: hypoxic ischemic encephalopathy.

Supplementary Tables

Supplementary Table 1: Data extraction table for scoping review.

Study Title	
First Author	
DOI	
Year of Publication	
Study Type	
Study Population	
Number All Included Patients	
Number Patients DCP	
Aetiology of CP included (Hypoxic Event/ Hyperbilirubinaemia/ Other)	
Age at inclusion	
GA (weeks)	
Birth Weight (g)	
Imaging Modality (Field+Sequence)	
Number of DCP with MRI	
Images Provided (y/n)	
Lesions assigned to DCP	
Systematic Lesion Classification	
Lesions Report (Table/Text)	
Outcome Structural Imaging	
Outcome CP Subtype	
Outcome Classification Scale (GMFCs, MACS, CFCS)	
Outcome Motor Scale (DIS, BFMDRS,…)	
Outcome Other (structural/functional/intervention)	
Video reported	

Supplementary Table 2: Summary of the 23 DCP cases identified from literature which have traceable imaging slices. When not given for individual cases, the age range of all DCP cases included in respective study is provided. Further clinical details are provided, when given in publication. SCPE refers to definition provided by Surveillance of Cerebral Palsy in Europe.

Paper	Cases	Figure	Age (years)	Sex	Diagnosis / Clinical Details	Lesion etiology
**Saini et al. 2021**[10]			1 - 4		Dyskinetic CP (SCPE)	
	Case #1	1	//	//	//	Hyperbilirubinemia
	Case #2	2	//	//	//	Perinatal asphyxia
**Kitai et al. 2021**[11]			0.6 - 20		Dyskinetic CP	
	Case #3	2A	//	//	//	Bilirubin encephalopathy
	Case #4	2B	//	//	//	Hypoxic-ischemic encephalopathy
**Park et al. 2014**[12]					Dyskinetic CP (SCPE)	
	Case #5	2B	33	male	GMFCS: 4<br>BADS: 18	//
	Case #6	2C	23	female	GMFCS: 2<br>BADS: 19	//
	Case #7	2D	35	male	GMFCS: 4<br>BADS: 22	//
	Case #8	2E (1-2)	16	female	GMFCS: 2<br>BADS: 19	//
**Yokochi et al. 1991**[13]					Athetotic CP	
	Case #9	1A-B	6	female	//	//
	Case #10	2	3	female	//	//
	Case #11	3	3	male	//	//
**Sun et al. 2018**[14]					Dyskinetic CP (SCPE)	
	Case #12	1 (upper)	1	female	Dystonic subtype	Hyperbilirubinemia
	Case #13	2 (mid)	8	male	Choreoathetotic subtype	Hypoxic-ischemic encephalopathy
**Derinkuyu et al. 2016**[15]						

	Case #14	2 (a-d)	3	female	Dyskinetic CP	Hypoxic-ischemic encephalopathy
**Laporta-Hoyos et al. 2018**[16]						
	Case #15	3	14	male	Dyskinetic CP (SCPE)	//
**Griffith et al. 2009**[17]			1 - 16			
	Case #16	3	//	//	Dyskinetic CP	Hypoxic-ischemic encephalopathy
**Koy et al. 2014**[18]					Dyskinetic CP	
	Case #17	1	22	//	General. dystonia + spasticity<br><br>GMFCS: 5<br>BFMDRS-M: 100<br>BFMDRS-D: 26	Peripartal asphyxia
**Gkoltsiou et al. 2008**[19]						
	Case #18		1	female	Mild dyskinetic CP, hearing loss	Hyperbilirubinemia (G6PD-deficiency)
**Cajigas et al. 2023**[20]						
	Case #19	1A	19	female	Dyskinetic CP (SCPE)<br>BFMDRS: 51	Perinatal hypoxia
**Okumura et al. 2006**[21]						
	Case #20	1	4	male	Athetoid CP	Kernicterus
**Yilmaz et al. 2002**[22]						
	Case #21	1	1.3	male	Dyskinetic CP and limitation of vertical gaze	Kernicterus
**Menkes et al. 1994**[23]					Extrapyramidal CP	
	Case #22	2	5	//	Spasticity, mental retardation	Hypoxic-ischemic encephalopathy
	Case #23	3 (A-D)	6	//	Spasticity	Perinatal asphyxia

Supplementary Table 3: Characteristics of studies included in the scoping review. Background blue for general literature characteristics, green for demographics of included patients, orange for imaging-related results and grey for non-imaging outcomes. Reference of studies with specific result given (according to Suppl. Table 4).

Study Characteristics	**Results of Scoping Review**	**Nr. Studies (%)**
Publication date	1991-2023	All
Study type	Observational (cross-sectional/populational) [1,3,5-8,10,11,15,18-21,26-31,33,35,41,48]	23 (48)
	Retrospective (not further assignable) [2,4,9,12,14,17,23,25,32,34,38]	11 (23)
	Prospective non-interventional [13,16,22,24]	4 (8)
	Case series [36,37,40,42,46]	5 (10)
	Case reports [39,43,44,47]	4 (8)
	Randomized controlled trial [45]	1 (2)
Total patients included	10147	-
Total DCP included	1870	-
Study population	Primarily patients with DCP [3,5-9,12,16-18,21-23,27,29,33,36,37,40,43-47]	24 (50)
	≥ 2 CP subtypes [1,2,4,10,11,13,15,19,20,25,28,30-32,35,38,48]	17 (35)
	Other (e.g. "childhood dystonia" or "children at risk for CP") [14,24,26,34,39,41,42]	7 (15)
Age at inclusion	Birth to 62 years	44 (92)
Sex	59,7% male	34 (71)
Gestational age	23-41 weeks	18 (38)
Birth weight	620-4500g	16 (33)
Cause of CP	HIE, BE and other [1,3,7,10,14,16-19,21,24,27,30,31,33,35]	16 (33)
	HIE only [26,32,36,39,41,45-47]	8 (17)
	BE only [12,23,34,40,42-44]	7 (15)
	HIE and BE [5,9]	2 (4)

	HIE and “other” [28,37,38]	3 (6)
	Cause not specified [2,4,6,8,11,13,15,20,22,25,29,48]	12 (25)
CP-subtype definition	According to SCPE guidelines [1-3,6-8,10,11,13,16,18,20-22,29-31,35]	17 (35)
	According to other definitions [11,38,46,48]	4 (8)
	Not specified	27 (56)
Imaging modality	Conventional MRI	42 (86)
	- Structural connectivity measures [2,4,8,16]	4 (8)
	- Functional connectivity measures (rs-fMRI) [13,22]	2 (4)
	MRI and [CT and/or ultrasound] [6,18,30,31,35,48]	6 (13)
Age at MRI	Day 20 – 62 years	24 (50)
Reporting of imaging results	Systematic established classification (e.g. MRICS) [1,3,6,8,11,18-20,27,29-32,35,38,45]	16 (33)
	Other (e.g. in extent, using unpublished patterns of interest)	32 (67)
Non-imaging outcomes	Functional classification scales (GMFCS, MACS, CFCS, other)	23 (48)
	- Only GMFCS [1-3,8,10,13,15,22,24,41,45,48]	12 (25)
	- GMFCS and [MACS and/or CFCS and/or other] [5-7,11,12,16,18,20,21,27,29]	11 (23)
	Quantification of motor symptoms using scores/scales	17 (35)
	- “Burke Fahn Marsden Dystonia Rating Scale” (BFMDRS) [7,9,14,24,33,37,45]	7 (15)
	- “Barry-Albright Dystonia Scale” (BADS) [3,8,14,18]	4 (8)
	- “Dyskinesia Impairment Scale” (DIS) [6,7,26,45]	4 (8)
	- “Alberta Infant Motor Scale” (AIMS) [33,41]	2 (4)
	Prevalence of CP subtype (dyskinetic/spastic/ataxic) in population [1,10,11,15,19,20,25,28,30,31,35,48]	12 (25)
	Non-motor outcomes (quality of life, activities of daily living, cognition) [9-11,16,21,22,27,29,33,34,38,41]	12 (25)
	Electrophysiological measures (motor evoked potentials, EMG, EEG) [7,8,24]	3 (6)
	Therapeutic intervention (DBS) [9,14,34,39,47]	5 (10)

Supplementary Table 4: Title, year of publication, first author and DOI of all papers included in the scoping review. Articles highlighted in grey were excluded from quantification of imaging findings since a) exact number of DCP patients receiving MRI was not reported (n=3)[1,11,15] or b) they only assessed a specific imaging feature, and it is not clear if other findings were absent or just not reported (n=5)[5,12,25,38,40].

Number	Title	Year of Publication	First Author	DOI
1	Which is the Most Common Physiologic Type of Cerebral Palsy?	2022	Kamate	https://doi.org/10.4103/0028-3886.349640
2	Anatomical characterization of athetotic and spastic cerebral palsy using an atlas-based analysis	2013	Yoshida	https://doi.org/10.1002/jmri.23931
3	Hyperbilirubinemia and Asphyxia in Children With Dyskinetic Cerebral Palsy	2021	Saini	https://doi.org/10.1016/j.pediatrneurol.2021.02.002
4	Athetotic and spastic cerebral palsy: anatomic characterization based on diffusion-tensor imaging	2011	Yoshida	https://doi.org/10.1148/radiol.11101783
5	Functional outcomes of children with dyskinetic cerebral palsy depend on etiology and gestational age	2021	Kitai	https://doi.org/10.1016/j.ejpn.2020.11.002
6	Clinical patterns of dystonia and choreoathetosis in participants with dyskinetic cerebral palsy	2015	Monbaliu	https://doi.org/10.1111/dmcn.12846
7	Using both electromyography and movement disorder assessment improved the classification of children with dyskinetic cerebral palsy	2022	Lorentzen	https://doi.org/10.1111/apa.16152
8	Neuroradiological and neurophysiological characteristics of patients with dyskinetic cerebral palsy	2014	Park	https://doi.org/10.5535/arm.2014.38.2.189
9	Pallidal stimulation for acquired dystonia due to cerebral palsy: beyond 5 years	2014	Romito	https://doi.org/10.1111/ene.12596
10	Children with dyskinetic cerebral palsy are severely affected as compared to bilateral spastic cerebral palsy	2019	Préel	https://doi.org/10.1111/apa.14806
11	Dyskinetic vs Spastic Cerebral Palsy: A Cross-sectional Study Comparing Functional Profiles, Comorbidities, and Brain Imaging Patterns	2018	Reid	https://doi.org/10.1177/0883073818776175
12	Diagnosis of Bilirubin Encephalopathy in Preterm Infants with Dyskinetic Cerebral Palsy	2020	Kitai	https://doi.org/10.1159/000502777

13	Functional Connectivity Alterations in Children with Spastic and Dyskinetic Cerebral Palsy	2018	Quin	https://doi.org/10.1155/2018/7058953
14	Pallidal stimulation in children: comparison between cerebral palsy and DYT1 dystonia	2013	Marks	https://doi.org/10.1177/0883073813488674
15	Clinical and MRI correlates of cerebral palsy: the European Cerebral Palsy Study	2006	Bax	https://doi.org/10.1001/jama.296.13.1602
16	Whole-brain structural connectivity in dyskinetic cerebral palsy and its association with motor and cognitive function	2017	Ballester-Plané	https://doi.org/10.1002/hbm.23686
17	Magnetic resonance imaging in athetotic cerebral palsied children	1991	Yokochi	https://doi.org/10.1111/j.1651-2227.1991.tb11955.x
18	Dyskinetic cerebral palsy: a population-based study of children born between 1991 and 1998	2007	Himmelmann	https://doi.org/10.1111/j.1469-8749.2007.00246.x
19	Magnetic resonance imaging, risk factors and co-morbidities in children with cerebral palsy	2010	Prasad	https://doi.org/10.1007/s00415-010-5782-2
20	The Origin of the Cerebral Palsies: Contribution of Population-Based Neuroimaging Data	2020	Horber	https://doi.org/10.1055/s-0039-3402007
21	Clinical characteristics and functional status of children with different subtypes of dyskinetic cerebral palsy	2018	Sun	https://doi.org/10.1097/md.0000000000010817
22	Aberrant Interhemispheric Functional Organization in Children with Dyskinetic Cerebral Palsy	2019	Qin	https://doi.org/10.1155/2019/4362539
23	Kernicterus in preterm infants	2009	Okumura	https://doi.org/10.1542/peds.2008-2791
24	EEG measures of sensorimotor processing and their development are<br>abnormal in children with isolated dystonia and dystonic cerebral palsy	2021	McClelland	https://doi.org/10.1016/j.nicl.2021.102569
25	A magnetic resonance imaging finding in children with cerebral palsy: Symmetrical central tegmental tract hyperintensity	2017	Derinkuyu	https://doi.org/10.1016/j.braindev.2016.10.004
26	Dyskinesia Impairment Scale scores in Dutch pre-school children after neonatal therapeutic hypothermia	2020	Kuiper	https://doi.org/10.1016/j.ejpn.2020.07.013
27	Brain lesion scores obtained using a simple semi-quantitative scale from MR imaging are associated with motor function, communication and cognition in dyskinetic cerebral palsy	2018	Laporta-Hoyos	https://doi.org/10.1016/j.nicl.2018.06.015

28	Regional cerebral glucose metabolism in clinical subtypes of cerebral palsy	1991	Kerrigan	https://doi.org/10.1016/0887-8994(91)90024-f
29	Prevalence, birth, and clinical characteristics of dyskinetic cerebral palsy compared with spastic cerebral palsy subtypes: A Norwegian register-based study	2023	Evensen	https://doi.org/10.1111/dmcn.15598
30	The panorama of cerebral palsy in Sweden part XII shows that patterns changed in the birth years 2007-2010	2018	Himmelmann	https://doi.org/10.1111/apa.14147
31	The changing panorama of cerebral palsy in Sweden. X. Prevalence and origin in the birth-year period 1999-2002	2010	Himmelmann	https://doi.org/10.1111/j.1651-2227.2010.01819.x
32	Anatomic localization of dyskinesia in children with "profound" perinatal hypoxic-ischemic injury	2010	Griffiths	https://doi.org/10.3174/ajnr.a1854
33	Young adults with dyskinetic cerebral palsy improve subjectively on pallidal stimulation, but not in formal dystonia, gait, speech and swallowing testing	2014	Koy	https://doi.org/10.1159/000360984
34	Serial brain MRI and ultrasound findings: relation to gestational age, bilirubin level, neonatal neurologic status and neurodevelopmental outcome in infants at risk of kernicterus	2009	Gkoltsiou	https://doi.org/10.1016/j.earlhumdev.2008.09.008
35	The panorama of cerebral palsy in Sweden part XIII shows declining prevalence in birth-years 2011-2014	2022	Himmelmann	https://doi.org/10.1111/apa.16548
36	Athetoid cerebral palsy with cysts in the putamen after hypoxic-ischaemic encephalopathy	1992	Rutherford	https://doi.org/10.1136/adc.67.7_spec_no.846
37	Cerebellar deep brain stimulation for the treatment of movement disorders in cerebral palsy	2023	Cajigas	https://doi.org/10.3171/2023.1.JNS222289
38	Bilateral lesions of thalamus and basal ganglia: origin and outcome	2002	Krägeloh-Mann	https://doi.org/10.1017/s0012162201002389
39	Brain magnetic resonance imaging in suspected extrapyramidal cerebral palsy: observations in distinguishing genetic-metabolic from acquired causes	1997	Hoon	https://doi.org/10.1016/s0022-3476(97)70160-4
40	Single photon emission computed tomography and serial MRI in preterm infants with kernicterus	2006	Okumura	https://doi.org/10.1016/j.braindev.2005.11.004
41	Motor testing at 1 year improves the prediction of motor and mental outcome at 2 years after perinatal hypoxic–ischaemicencephalopathy	2009	van Schie	https://doi.org/10.1111/j.1469-8749.2009.03302.x

42	Magnetic resonance imaging in three children with kernicterus	2001	Sugama	https://doi.org/10.1016/S0887-8994(01)00306-X
43	Thalamic involvement in a patient with kernicterus	2002	Yilmaz	https://doi.org/10.1007/s003300100993
44	Proton magnetic resonance spectroscopic images in preterm infants with bilirubin encephalopathy	2012	Kamei	https://doi.org/10.1016/j.jpeds.2011.09.036
45	Quality of Life After Deep Brain Stimulation of Pediatric Patients with Dyskinetic Cerebral Palsy: A Prospective, Single-Arm, Multicenter Study with a Subsequent Randomized Double-Blind Crossover (STIM-CP)	2023	Koy	https://doi.org/10.1002/mds.28898
46	Clinical and MR correlates in children with extrapyramidal cerebral palsy.	1994	Menkes	PMID: 8197940 (DOI not available)
47	Bilateral Thalamic Lesions in a Newborn with Intrauterine Asphyxia After Maternal Cardiac Arrest — a Case Report with Literature Review	2001	Banerjea	https://doi.org/10.1038/sj.jp.7210560
48	Population-based study of neuroimaging findings in children with cerebral palsy	2011	Towsley	https://doi.org/10.1016/j.ejpn.2010.07.005

References (Supplementary Material)

1. Al-Fatly B, Giesler SJ, Oxenford S, et al. Neuroimaging-based analysis of DBS outcomes in pediatric dystonia: Insights from the GEPESTIM registry. *NeuroImage Clin*. 2023;39:103449. doi:10.1016/J.NICL.2023.103449

2. Molfese PJ, Glen D, Mesite L, et al. The Haskins pediatric atlas: a magnetic-resonance-imaging-based pediatric template and atlas. *Pediatr Radiol*. 2021;51(4):628-639. doi:10.1007/S00247-020-04875-Y/TABLES/3

3. Cacciola A, Milardi D, Bertino S, et al. Structural connectivity-based topography of the human globus pallidus: Implications for therapeutic targeting in movement disorders. *Mov Disord*. 2019;34(7):987-996. doi:10.1002/MDS.27712

4. Friston KJ, Holmes AP, Worsley KJ, Poline J -P, Frith CD, Frackowiak RSJ. Statistical parametric maps in functional imaging: A general linear approach. *Hum Brain Mapp*. 1994;2(4):189-210. doi:10.1002/HBM.460020402

5. Avants BB, Epstein CL, Grossman M, Gee JC. Symmetric diffeomorphic image registration with cross-correlation: Evaluating automated labeling of elderly and neurodegenerative brain. *Med Image Anal*. 2008;12(1):26-41. doi:10.1016/J.MEDIA.2007.06.004

6. Ewert S, Plettig P, Li N, et al. Toward defining deep brain stimulation targets in MNI space: A subcortical atlas based on multimodal MRI, histology and structural connectivity. *Neuroimage*. 2018;170:271-282. doi:10.1016/j.neuroimage.2017.05.015

7. Fonov V, Evans AC, Botteron K, Almli CR, McKinstry RC, Collins DL. Unbiased average age-appropriate atlases for pediatric studies. *Neuroimage*. 2011;54(1):313-327. doi:10.1016/J.NEUROIMAGE.2010.07.033

8. Bertino S, Basile GA, Bramanti A, et al. Spatially coherent and topographically organized pathways of the human globus pallidus. *Hum Brain Mapp*. 2020;41(16):4641-4661. doi:10.1002/HBM.25147

9. Tsagkaris S, Yau EKC, McClelland V, et al. Metabolic patterns in brain 18F-fluorodeoxyglucose PET relate to aetiology in paediatric dystonia. *Brain*. 2023;146(6):2512-2523. doi:10.1093/BRAIN/AWAC439

10. Saini AG, Sankhyan N, Malhi P, Ahuja C, Khandelwal N, Singhi P. Hyperbilirubinemia and Asphyxia in Children With Dyskinetic Cerebral Palsy. *Pediatr Neurol*. 2021;120:80-85. doi:10.1016/J.PEDIATRNEUROL.2021.02.002

11. Kitai Y, Hirai S, Okuyama N, et al. Functional outcomes of children with dyskinetic cerebral palsy depend on etiology and gestational age. *Eur J Paediatr Neurol*. 2021;30:108-112. doi:10.1016/J.EJPN.2020.11.002

12. Park BH, Park SH, Seo JH, Ko MH, Chung GH. Neuroradiological and Neurophysiological Characteristics of Patients With Dyskinetic Cerebral Palsy. *Ann Rehabil Med*. 2014;38(2):189-199. doi:10.5535/ARM.2014.38.2.189

13. Yokochi K, Aiba K, Kodama M, Fujimoto S. Magnetic resonance imaging in athetotic cerebral palsied children. *Acta Paediatr Scand*. 1991;80(8-9):818-823. doi:10.1111/j.1651-2227.1991.tb11955.x

14. Sun D, Wang Q, Hou M, et al. Clinical characteristics and functional status of children with different subtypes of dyskinetic cerebral palsy. *Med (United States)*. 2018;97(21). doi:10.1097/MD.0000000000010817

15. Derinkuyu BE, Ozmen E, Akmaz-Unlu H, Altinbas NK, Gurkas E, Boyunaga O. A magnetic resonance imaging finding in children with cerebral palsy: Symmetrical central tegmental tract hyperintensity. *Brain Dev*. 2017;39(3):211-217. doi:10.1016/J.BRAINDEV.2016.10.004

16. Laporta-Hoyos O, Fiori S, Pannek K, et al. Brain lesion scores obtained using a simple semi-quantitative scale from MR imaging are associated with motor function, communication and cognition in dyskinetic cerebral palsy. *NeuroImage Clin*. 2018;19:892-900. doi:10.1016/j.nicl.2018.06.015

17. Griffiths PD, Radon MR, Crossman AR, Zurakowski D, Connolly DJ. Anatomic Localization of Dyskinesia in Children with "Profound" Perinatal Hypoxic-Ischemic Injury. *Am J Neuroradiol*. 2010;31(3):436-441. doi:10.3174/AJNR.A1854

18. Koy A, Pauls KAM, Flossdorf P, et al. Young Adults with Dyskinetic Cerebral Palsy Improve Subjectively on Pallidal Stimulation, but not in Formal Dystonia, Gait, Speech and Swallowing Testing. *Eur Neurol*. 2014;72(5-6):340-348. doi:10.1159/000360984

19. Gkoltsiou K, Tzoufi M, Counsell S, Rutherford M, Cowan F. Serial brain MRI and ultrasound findings: Relation to gestational age, bilirubin level, neonatal neurologic status and neurodevelopmental outcome in infants at risk of kernicterus. *Early Hum Dev*. 2008;84(12):829-838. doi:10.1016/J.EARLHUMDEV.2008.09.008

20. Cajigas I, Morrison MA, Luciano MS, Starr PA. Cerebellar deep brain stimulation for the treatment of movement disorders in cerebral palsy. *J Neurosurg*. 2023;139(3):605-614. doi:10.3171/2023.1.JNS222289

21. Okumura A, Hayakawa F, Maruyama K, Kubota T, Kato K, Watanabe K. Single photon emission computed tomography and serial MRI in preterm infants with kernicterus. *Brain Dev*. 2006;28(6):348-352. doi:10.1016/J.BRAINDEV.2005.11.004

22. Yilmaz Y, Ekinci G. Thalamic involvement in a patient with kernicterus. *Eur Radiol*. 2002;12(7):1837-1839. doi:10.1007/S003300100993/METRICS

23. Menkes JH, Curran J. Clinical and MR Correlates in Children with Extrapyramidal Cerebral Palsy. Published online 1994.
